# Recent advances in functional assays of WRKY transcription factors in plant immunity against pathogens

**DOI:** 10.3389/fpls.2024.1517595

**Published:** 2025-01-23

**Authors:** Wenjing Wang, Haihui Cao, Jiahao Wang, Hongbo Zhang

**Affiliations:** ^1^ Tobacco Research Institute, Chinese Academy of Agricultural Sciences, Qingdao, China; ^2^ College of Agronomy, Qingdao Agricultural University, Qingdao, China

**Keywords:** WRKY TFs, biotic stress, plant resistance, defense signaling, plant-pathogen interactions

## Abstract

WRKY transcription factors (TFs) are one of the largest transcription factor families in plants and play important roles in plant processes, most notably in responding to diverse biotic and abiotic stresses. This article reviews the recent research progresses on WRKY TFs in regulating plant immunity, which includes both positive and negative regulation. WRKY TFs were shown to regulate plant defense against pathogens including fungi, bacteria, oomycetes, and viruses by modulating downstream pathogen resistance genes or interacting with other regulators. Plant signaling pathways or components involved in the regulatory network of WRKY-mediated plant immunity mainly involve the action of phytohormones, MAPKs (Mitogen-activated protein kinases), and other transcription factors. The interaction of WRKY TFs with these factors during pathogen resistance was discussed in this article, which may contribute to understanding the mechanisms of WRKY transcription factors in plant immunity.

## Introduction

WRKY transcription factors (TFs) compose one of the largest transcription factor families in plants, and they play important roles in plant growth, development, and stress tolerance (Rushton et al., 2010). The first WRKY gene was isolated from sweet potato and named as *SPF1* (sweet potato factor 1) in 1994 (Ishiguro and Nakamura, 1994). Subsequently, a vast amount of WRKY TFs (**Supplementary Table S1**) have been identified in various plant genera as well as non-plant species, such as fungi, diplomonads, and amoebae (Zhang and Wang, 2005; Rinerson et al., 2015). In the non-plant species *Dictyostelium discoideum* and *Giardia lamblia*, only one WRKY protein was identified, whereas many WRKY proteins were identified in flowering-plants, such as *Brassica napus* and *Zea mays* containing 153 and 180 WRKY proteins, respectively. Researchers proposed that the evolution of WRKY TFs includes early lateral gene transfer to non-plant organisms (Rinerson et al., 2015).

The most typical feature of WRKY TFs is the conserved N-terminal amino acid sequence WRKYGQK (Eulgem et al., 2000). In addition, they possess a typical zinc-finger structure at the C-terminus, with the sequence of CX4-5CX22-23HXH or CX7CX23HXC (Eulgem et al., 2000). In a few of WRKY proteins, the amino acid sequence WRKYGQK is replaced by WRRY, WSKY, WKRY, WVKY, or WKKY (Eulgem et al., 2000). In higher plants, the WRKY family has been divided into groups I, IIa, IIb, IIc, IId, IIe, and III based on their phylogenetic relationship and was proven to regulate the expression of downstream genes by binding to the W-box (TTGACC/T) in their promoters (Rushton et al., 2010). Thus, all genes, including WRKY genes containing W-box elements in the promoter, are assumed as the regulatory targets of WRKY TFs.

Over the past few decades, extensive and in-depth research has been conducted on WRKY TFs, which revealed the key roles of WRKY TFs in regulating plant growth, development, and stress response (Wang et al., 2022a; Khoso et al., 2022). Several previous articles have summarized the roles of WRKY TFs in regulating plant tolerance to biotic stresses (Eulgem and Somssich, 2007; Pandey and Somssich, 2009; Amorim et al., 2016; Finatto et al., 2018). In recent years, many novel works on the function of WRKY TFs in regulating plant resistance to biotic stresses have been reported. The current article summarizes recent progresses on the functions of WRKY TFs in regulating plant defense to pathogens including fungi, bacteria, oomycetes, and viruses, and aims to provide a comprehensive and systematic understanding of the regulatory network of WRKY TFs.

## Role of WRKY TFs in plant resistance to fungal pathogens

### Pathogens causing powdery mildew disease

**Figure 1:**
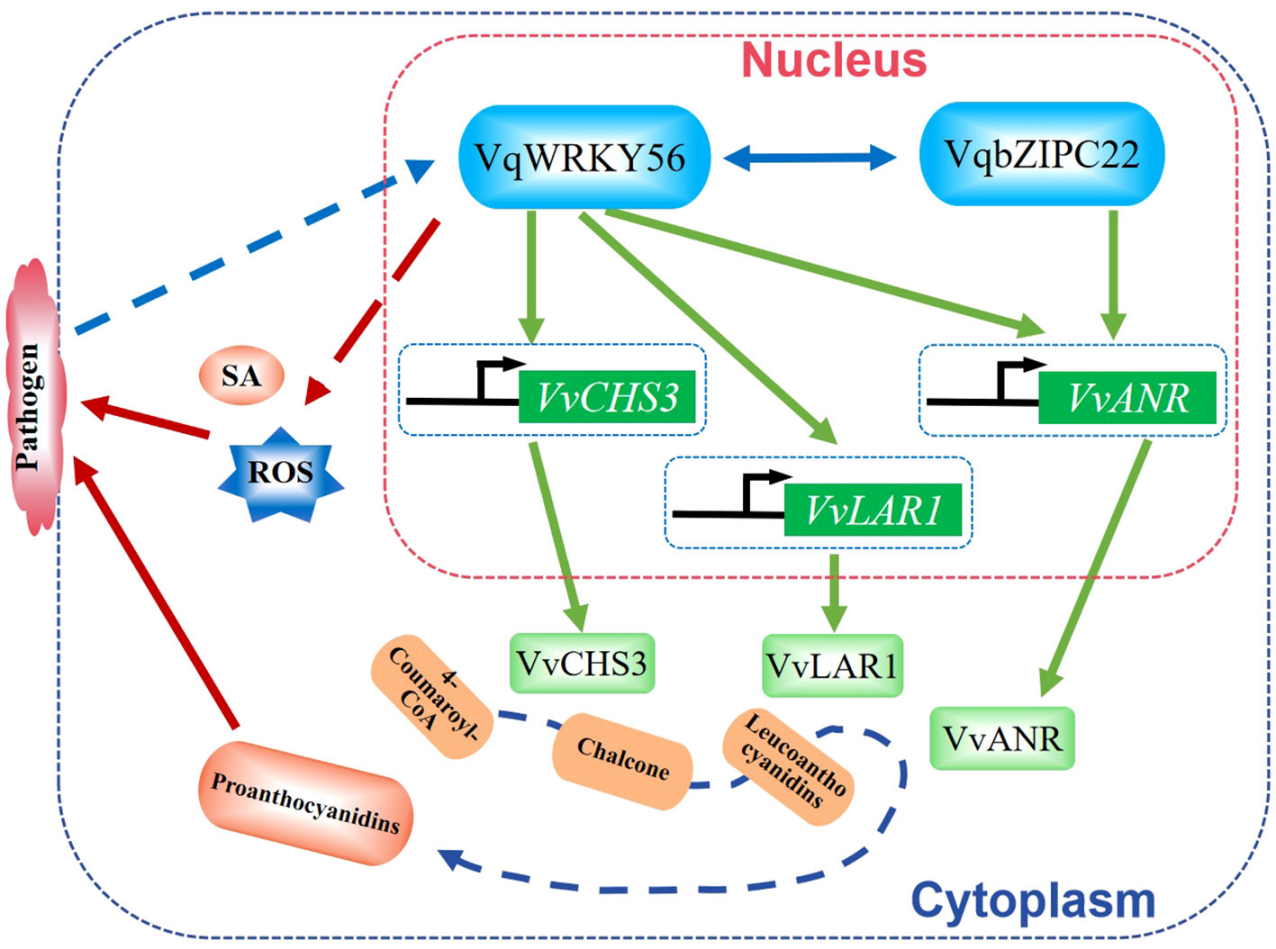
Schematic diagram of VqWRKY56 in regulating grape resistance against powdery mildew pathogen *Erysiphe necator*. VqWRKY56 interacts with VqbZIPC22 to activate the expression of PA (proanthocyanidin) biosynthetic genes *VvCHS3*, *VvLAR1*, and *VvANR* to promote the accumulation of PAs. The overexpression of VqWRKY56 can increase the accumulation of PAs, ROS, and SA, which provide resistance to powdery mildew pathogen *E. necator* (Wang et al., 2023a).

Powdery mildew is a fungal disease caused by several different genera of fungi and affects many plant species, such as grape, wheat, and barley, to cause huge losses to agricultural production. In grape, VqWRKY56, VqWRKY31, VqWRKY6, and VqWRKY52 were shown to regulate the resistance to powdery mildew pathogen *Erysiphe necator* (Wang et al., 2017a; Yin et al., 2022; Zhang et al., 2022; Wang et al., 2023a). Overexpression of VqWRKY56 increased resistance to powdery mildew by increasing the accumulation of proanthocyanidins (PAs), reactive oxygen species (ROS), and salicylic acid (SA). VqWRKY56 could interact with transcript factor VqbZIPC22 and activate the expression of PA biosynthetic genes *VvCHS3* (chalcone synthase 3), *VvLAR1* (leucoanthocyanidin reductase 1), and *VvANR* (anthocyanidin reductase) to increase the accumulation of PAs (Wang et al., 2023a). VqWRKY56 binds to the promoters of *VvCHS3*, *VvLAR1*, and *VvANR*, while VqbZIPC22 binds to the promoter of *VvANR* during this process (Wang et al., 2023a). Co-expression of *VqWRKY56* and *VqbZIPC22* significantly increased the transcription levels of *VvCHS3*, *VvLAR1*, and *VvANR* in grapes to enhance the resistance to powdery mildew pathogen (Wang et al., 2023a). These findings revealed a complex and elaborate mechanism by which WRKY TFs regulate grape resistance against *E. necator* (**Figure 1**).

WRKY TFs may regulate plant resistance to powdery mildew pathogen via various other manners. For instance, VqWRKY31 activates the SA signaling and promotes the biosynthesis of secondary metabolites, such as stilbenes, flavonoids, and proanthocyanidins, to enhance mildew pathogen resistance (Yin et al., 2022). *In vitro* assays demonstrated that VqWRKY31 directly binds to the promoters of *STS9* and *STS48*, two structural genes associated with stilbene synthesis, and activates their expression to regulate the resistance to mildew pathogen (Yin et al., 2022). VqWRKY6 interacts with VqbZIP1 to form a transcription factor complex and to activate the jasmonic acid (JA) signaling pathway, which enhances the accumulation of ROS and the expression of pathogenesis-related (PR) genes *PR3* and *PR4* to ultimately achieving the resistance against powdery mildew pathogens (Zhang et al., 2022). When ectopically expressed in *Arabidopsis thaliana*, VqWRKY52 positively regulates the resistance to powdery mildew pathogen through the SA signaling, but the underlying molecular mechanisms is yet to be revealed (Wang et al., 2017a). Similarly, ShWRKY81 positively regulates tomato resistance to powdery mildew pathogens by regulating the expression of SA-dependent genes and *PR* genes as well as the accumulation of hydrogen peroxide (H_2_O_2_) (Wang et al., 2023b). On the contrary, barley HvWRKY2 and wheat TaWRKY76-D negatively regulate host resistance to powdery mildew pathogens. HvWRKY2 targets a putative chitin receptor gene *HvCEBiP* and TaWRKY76-D interacts with a CC-NBS-LRR receptor protein Pm2b to promoting pathogen infection (Yu et al., 2023; Jin et al., 2022). These findings showed that WRKY TFs could affect plant resistance to pathogens causing powdery mildew diseases by altering phytohormone signaling, hydrogen peroxide production, or the function of receptor protein.

### Penicillium digitatum

*Penicillium digitatum* is a fungus causing green mold disease, one of the most challenging and destructive diseases of citrus worldwide. This disease accounts for approximately 60~90% of postharvest diseases in citrus. So far, four WRKY TFs have been isolated from citrus, including CsWRKY23, CsWRKY25, CsWRKY65, and CsWRKY70, and all these WRKY TFs have been reported to positively regulate resistance to *P. digitatum*. Specifically, CsWRKY23 promotes the accumulation of SA and ROS and enhances the thickness of cell walls to improve pathogen resistance (Wang et al., 2023c). It could directly target 48 genes, most of which are associated with plant-pathogen interaction, phytohormone signaling, secondary metabolite biosynthesis and other metabolisms (Wang et al., 2023c). Similar functions were observed for CsWRKY25 and CsWRKY65 in regulating pathogen resistance, during which CsWRKY25 activates the expression of target genes *RbohB* (respiratory burst oxidase homolog protein), *RbohD*, and *PR10* and CsWRKY65 activates the expression of *CsRbohB*, *CsRbohD*, *CsCDPK33* (calcium dependent protein kinase), and *CsPR10* (Wang et al., 2021a, b). CsWRKY70 activates the expression of a gene encoding salicylate carboxymethyltransferase (SAMT) involved in the SA biosynthesis and enhances host resistance to *P. digitam* (Deng et al., 2020). These reports indicated a complicated network of WRKY TFs in regulating citrus resistance to *P. digitatum*.

### Fusarium solani

*Fusarium solani* is a soil-borne pathogen known to cause apple replant disease (ARD) in all major apple-growing regions worldwide. This pathogen hinders tree growth and reduces the yield and quality of apple (Xiang et al., 2022). Researches show that the WRKY TFs MdWRKY75 and MdWRKY74 positively regulate apple resistance to *F. solani* (Xiang et al., 2022; Liu et al., 2023). MdWRKY75 promotes the accumulation of lignin to enhance apple resistance to *F. solani* via the MdWRKY75-MdERF114-MdMYB8-MdPRX63 complex, while MdWRKY74 alone binds to the promoter of *MdECHT* (endochitinase gene) and enhances chitin accumulation to improve pathogen resistance (Xiang et al., 2022; Liu et al., 2023). Consistently, PnWRKY9 in *Panax notoginseng* (Burk) F.H. Chen, a rare and valuable Chinese herb, regulates resistance to the root rot caused by *F. solani*, which severely affects the yield and quality of this herb (Zheng et al., 2022). During this process, PnWRKY9 regulates JA signaling and binds to the promoter of *PnDEFL1*, a JA-responsive and *F. solani* resistance-associated gene, to obtain pathogen resistance (Zheng et al., 2022). On the contrary, sugarcane ScWRKY3, which is upregulated by ABA but downregulated by SA and MeJA, negatively regulates plant resistance against *F. solani* infection (Wang et al., 2018).

### Fusarium oxysporum

*Fusarium oxysporum* is also a soil-borne pathogenic fungus with a wide range of hosts worldwide. It causes wilt diseases in more than 100 plants species, such as cotton, melons, bananas, legumes, and flowers etc (Wang et al., 2022b, 2022). In cotton, the group IIc WRKY TFs induce the GhMKK2-GhNTF6 pathway of mitogen-activated protein kinase (MAPK) cascade and increase the resistance to *F. oxysporum* by upregulating GhMYC2-mediated expression of flavonoid biosynthetic genes to promote the accumulation of flavonoids (Wang et al., 2022b). Similarly, PnWRKY22 of *P. notoginseng* upregulates SA levels to increase the resistance to *F. oxysporum* (Ning et al., 2021). In lily, LrWRKY2 and LrWRKY3 positively regulate the resistance to root rot, the most destructive disease caused by *F. oxysporum* (Li et al., 2021; Wang et al., 2022c). LrWRKY2 binds to the W-box in the promoter of *LrCHI2* (a chitinase-encoding gene) and activates its expression to accentuate defense responses (Li et al., 2021). On the other hand, LrWRKY3 targets the antimicrobial peptide-encoding gene *LrDef1* to increase pathogen resistance (Wang et al., 2022c). Overexpression of *LrWRKY3* in tobacco increased the transcription of JA biosynthesis-related genes and the SA-mediated responses to modulate pathogen resistance (Wang et al., 2022c). In tomato, *F. oxysporum* causes the extremely destructive soil-borne disease Fusarium crown and root rot (FCRR). Transcriptome analysis showed that *F. oxysporum* infection induces the MAPK signaling to stimulate tomato WRKY genes and initiate the resistance reaction to FCRR (Sun et al., 2022a).

### Alternaria alternata

*Alternaria alternata* is a fungus causing leaf spot and blight in many plants. Various WRKY TFs are known to regulate plant resistance to *A. alternata*. In *Populus nigra*, PsnWRKY70 enhances the resistance to *A. alternata* by activating the MAP kinase cascade genes, calcium channel protein-encoding genes, and calcium-dependent protein kinase genes (Wang et al., 2022d). It also directly binds to the promoters of homologous WRKY genes (such as *WRKY6*, *WRKY18*, *WRKY22*, and *WRKY22*-1) and those of LRR domain protein genes (such as LRR8, LRR-RLK, ADR1-like 2, and NB-ARC) to promote their expression and to enhance pathogen resistance (Wang et al., 2022d). On the contrary, apple MdWRKY120 negatively regulates the resistance to *A. alternata* in an unknown regulatory mechanism (Liu et al., 2022). Tobacco NaWRKY3 is a fine-tuned master regulator of the defense network against *A. alternata* by enhancing the expression of target genes involved in phytoalexin biosynthesis and the immune signaling conducted by phytohormones (JA and ET), ROS, and the *A. alternata* resistance long noncoding RNA *L2* and berberine bridge-like gene *NaBBL28* (Xu et al., 2023).

### Colletotrichum gloeosporioides

The fungus *Colletotrichum gloeosporioides* causes walnut anthracnose, a catastrophic disease adversely affecting the walnut production in China (Zhou et al., 2022). JrWRKY21 in walnut positively regulates the resistance to *C. gloeosporioides* by interacting with PTI5L, the transcriptional activator of *PR* gene, to form a WRKY21-PTI5L protein complex that activates the expression of *JrPR5L* (Zhou et al., 2022 *)*.

### *Puccinia horiana* Henn

The fungus *Puccinia horiana* Henn causes chrysanthemum white rust (CWR), which impairs the production and ornamental value of chrysanthemum. Recent study showed that CmWRKY15-1 positively regulates chrysanthemum defense response to *P. horiana* (Gao et al., 2022). CmWRKY15-1 interacts with CmNPR1 (non-expressor of pathogenesis-related gene 1) to activate the expression of downstream *PR* genes and enhance resistance through the SA pathway (Gao et al., 2022).

### Botrytis cinerea

*Botrytis cinerea* is a fungus with a wide range of hosts and can infect seedlings, fruits, and storage products of plants, causing heavy losses in agricultural production (Fu et al., 2022). Several studies in Arabidopsis have reported the regulation of *B. cinerea* resistance by WRKY TFs, such as AtWRKY33, AtWRKY2/AtWRKY34 (close homologs of AtWRKY33), AtWRKY3, AtWRKY4, AtWRKY18, AtWRKY40, AtWRKY60, and AtWRKY70. A few articles have comprehensively discussed the regulatory roles of these WRKY TFs (Amorim et al., 2016; Chen et al., 2019).

Studies in *Lillium. regale* revealed that LrWRKY39, LrWRKY4, and LrWRKY12 positively regulate the immune response to *B. cinerea*, while LrWRKY41a was identified to play a negative role (Cui et al., 2018a; Fu et al., 2022). The distinct functions of these WRKY members during *Botrytis* resistance may be related to the transcriptional changes in SA- and JA-responsive genes. Consistently, tomato SlWRKY46 and SpWRKY6 play negative roles during *B. cinerea* infection by inhibiting the activities of antioxidant and defense-related enzymes that influence the SA- and JA-signaling, ROS homeostasis, and the biosynthesis of SA, JA, and ABA (Liu et al., 2016; Shu et al., 2021). In contrast, FvWRKY50 in woodland strawberry positively regulates the resistance to *B. cinerea* by adjusting the JA signaling and the expression of defense-related genes (Ma et al., 2021). In rubber (*Hevea brasiliensis*), HbWRKY40 positively regulates host resistance to *B. cinerea* (Yang et al., 2020). Notably, overexpression of *HbWRKY40* significantly induced the reactive oxygen burst in *Nicotiana benthamiana* and increased the resistance against *B. cinerea* in Arabidopsis via regulating multiple downstream genes involved in pathogen resistance (Yang et al., 2020). Conversely, overexpression of *PtrWRKY73* from *Populus trichocarpa* in Arabidopsis increased the sensitivity to *B. cinerea*, accompanied by reduced expression of the defense-related gene *PAL4* and upregulation of the SA-mediated defense-associated genes *PR1*, *PR2*, and *PAD4* (Duan et al., 2015). Accordingly, GbWRKY1 of cotton negatively regulates the resistance to *B. cinerea* and *V. dahliae* by activating the expression of JA signaling suppressor gene *GhJAZ1* to attenuate the JA responses (Li et al., 2014).

### Rhizopus stolonifer

Peach PpWRKY45 and PpWRKY70 have been demonstrated to regulate the resistance against *Rhizopus stolonifer*, which causes severe fruit rot disease (Ji et al., 2021a, b; Ji et al., 2022; Li et al., 2022a). PpWRKY46 and PpWRKY53 contribute to methyl jasmonate (MeJA)-primed defense by regulating the energy metabolism-related genes *PpSDH* and *PpCOX15*, while PpWRKY22 physically interacts with PpHOS1/PpTGA1 (PpHOS1: a RING E3 ubiquitin ligase; TGA1: transcript factor) to regulate several SA-responsive *PR* genes and to positively modulate pathogen resistance (Ji et al., 2022; Li et al., 2022a). Accordingly, PpWRKY45 and PpWRKY70 participate in the MeJA-primed defense response against *R. stolonifera*, which occurs via the activation of JA biosynthetic genes, *PR* genes, and those of phenylpropanoid metabolism (Ji et al., 2021a, b).

### Zymoseptoria tritici

Septoria tritici blotch (STB) caused by the fungus *Zymoseptoria tritici* is currently the main threat to wheat production in the temperate zone. Recent studies have revealed the role of WRKY TFs in regulating wheat responses to this fungus. For instance, TaWRKY10, a novel modulator of JA responses, negatively regulates the expression of JA receptor gene *TaCOI1* to affect JA response and resistance to *Z. tritici* (Campanaro et al., 2021).

### Botryosphaeria dothidea

In apple, *Botryosphaeria dothidea* causes various diseases, which adversely affect yield. Several transcription factors regulating host responses against this pathogen have been identified. Particularly, MdWRKY15 plays a positive role in regulating the resistance to *B. dothidea* by activating the expression of *MdICS1*, encoding an isochorismate synthase that is essential in SA accumulation, to increase the SA accumulation and the expression of various disease response-related genes (Zhao et al., 2020).

### Verticillium dahliae

The wilt caused by *Verticillium dahliae* is a destructive disease, resulting in severe yield and quality losses in cotton worldwide. GhWRKY41, GhWRKY1-like and GhWRKY70 are associated with wilt resistance in cotton (Xiong et al., 2019; Hu et al., 2021; Xiao et al., 2023). Among these WRKY TFs, GhWRKY41 physically interacts with itself and directly activates its transcription (Xiao et al., 2023). The GhWRKY41 homodimer further activates the expression of *GhC4H* (cinnamate 4-hydroxylase) and *Gh4C*L(cinnamate 4-hydroxylase) to influence the phenylpropanoid metabolism and to modulate the accumulation of lignin and flavonoid (Xiao et al., 2023). GhWRKY41 also forms a positive feedback regulatory loop and improves cotton defense response against *V. dahliae* by regulating the phenylpropanoid metabolism (Xiao et al., 2023). GhWRKY1-like positively regulates cotton resistance to *V. dahliae* via directly manipulating the lignin biosynthesis. GhWRKY1-like can interact with the lignin biosynthesis related genes *GhPAL6* and *GhCOMT1* and activates the expression of *GhPAL6* and *GhCOMT1* to enhance the biosynthesis of total lignin (Hu et al., 2021). On the other hand, GhWRKY70 negatively regulates cotton resistance to *V. dahliae* through its effect on the biosynthesis and associated signaling of phytohormones ET, JA, and SA (Xiong et al., 2019).

### Colletotrichum musae

*Colletotrichum musae* is a major pathogen known to infect plant species of the *Musa* genus. Studies in banana has shown that WRKY TFs can take part in the plant defense regulation conducted by NACs (NAM/ATAF/CUC), a plant-specific transcription factor family that have received much attention. In banana, the NAC transcription factor MaNAC5 cooperates with MaWRKY1 and MaWRKY2 to activate the transcriptional activities of *MaPR1*-*1*, *MaPR2*, *MaPR10c*, and *MaCHIL1* (chitinase-like 1) to regulate SA- and MeJA-induced resistance to *C. musae* (Shan et al., 2016).

### Sclerotinia sclerotiorum

*Sclerotinia sclerotiorum* is a necrotrophic fungal pathogen causing the stem rot of *Brassica napus*, a major oil crop worldwide. *B. napus* BnWRKY33 positively regulates the resistance to *S. sclerotiorum* by enhancing the expression of genes involved in camalexin biosynthesis and those regulated by SA and JA (Liu et al., 2018a). In contrast, BnWRKY15 increases *B. napus* susceptibility to *S. sclerotiorum* by interacting with BnWRKY33 and inhibiting its transcriptional activation (Liu et al., 2018a).

### *Puccinia striiformis* f. sp. *tritici*

The stripe rust caused by *Puccinia striiformis* f. sp. *tritici* (Pst) is a destructive disease of wheat. Studies have reported diverse roles of wheat TaWRKY49 and TaWRKY62 in the defense response against stripe rust (Wang et al., 2017b). TaWRKY49 negatively regulates wheat resistance to Pst by increasing the expression of SA- and JA-responsive genes *TaPR1.1* and *TaAOS* and ROS-associated genes *TaCAT* and *TaPOD* and decreasing the expression of ethylene-responsive gene *TaPIE1* (Wang et al., 2017b). In contrast, TaWRKY62 positively regulates wheat resistance to Pst by differentially controlling SA, JA, ET, and ROS mediated signaling (Wang et al., 2017b). TaWRKY19 could bind to a W-box element in the promoter of *TaNOX10*, which encodes an NADPH oxidase and is required for ROS generation, and repress ROS generation, resulting the decreased host resistance to Pst (Wang et al., 2022e).

In barley, the WRKY TFs HvWRKY6 and HvWRKY70 positively regulate the resistance to Pst and *Blumeria graminis* f. sp. *tritici* (Bgt) infection. Notably, overexpression of *HvWRKY6* in barley improved resistance to leaf rust (*Puccinia triticina*, Pt), Fusarium crown rot (FCR, *Fusarium pseudograminearum*, Fpg), and sharp eyespot (*Rhizoctonia cerealis*, Rc) by activating the SA signaling and suppressing the ABA and JA signaling (Li et al., 2020; 2022b). The findings of earlier studies indicated that HvWRKY6 could offer a broad-spectrum resistance against upto five fungal pathogens.

### Magnaporthe oryzae

The blast fungus *Magnaporthe oryzae* is one of the most destructive pathogens of rice. Studies have shown that many WRKY factors play regulatory roles in rice resistance against *M. oryzae*, including OsWRKY13, OsWRKY22, OsWRKY24, OsWRKY28, OsWRKY30, OsWRKY31, OsWRKY42, OsWRKY45-1, OsWRKY45-2, OsWRKY47, OsWRKY53, OsWRKY62, OsWRKY76, and OsWRKY89. These WRKY TFs, except for OsWRKY28, OsWRKY42, and OsWRKY76, positively regulate rice resistance to blast disease (Wang et al., 2007; Qiu et al., 2007; Peng et al., 2008; Chujo et al., 2013; Cheng et al., 2015; Yokotani et al., 2018; Xu et al., 2022).

The OsWRKY45-2-OsWRKY13-OsWRKY42 transcriptional regulatory cascade play important roles in rice resistance to *M. oryzae*. OsWRKY42 functions downstream of OsWRKY13 and OsWRKY45-2 to negatively regulate rice resistance to *M. oryzae* by directly or indirectly suppressing the JA biosynthetic genes and JA-dependent defense signaling, and can be directly suppressed by OsWRKY13 (Cheng et al., 2015). Consistently, the overexpression of *OsWRKY28* enhanced rice susceptibility to *M. oryzae* (Chujo et al., 2013).

In contrast, OsWRKY89 contributes positively to the resistance against blast by regulating the wax content/deposition on leaf surface (Wang et al., 2007). The rice knockdown lines of *OsWRKY89* showed reduced wax content and increased susceptibility to *M. grisea* (Wang et al., 2007). OsWRKY53 also positively regulates blast resistance, and its function is activated by OsMPK3/OsMPK6-mediated phosphorylation (Chujo et al., 2014). The regulation of rice resistance against *M. oryzae* by OsWRKY30 is related to the activation and expression of JA biosynthetic genes *LOX* and *AOS2* and *PR* genes *PR3* and *PR10* (Peng et al., 2012). OsWRKY31 acts downstream of OsMKK10-2 in the OsMKK10-2–OsMPK3–OsWRKY31 cascade as a key regulator of rice defense to *M. oryzae*. OsMKK10-2 enhances the resistance against *M. oryzae* through increasing the JA and SA accumulation and decreasing the indole-3-acetic acid level (Wang et al., 2023d). Modification of OsWRKY31 by phosphorylation and ubiquitination is involved in the OsMKK10-2-mediated defense pathway (Wang et al., 2023d).

The WRKY TFs reported to regulate plant resistance against fungal pathogens are shown in **Table 1**.

**Table 1:** The list of WRKY TFs that regulate plant responses to fungal pathogens.

Pathogens	Names	Species	Target proteins	Functions	References
*Erysiphe necator*	VqWRKY56	grapevine	VqbZIPC22, related with proanthocyanidin	positive	Wang et al., 2023a
	VqWRKY31	grapevine	STS9 and STS48, related with SA, ROS, and stilbene	positive	Yin et al., 2022
	VqWRKY6	grapevine	VqbZIP1, related with JA, ROS, PR1, PR4	positive	Zhang et al., 2022
	VqWRKY52	grapevine	related with SA	positive	Wang et al., 2017a
*Oidium neolycopersici*	ShWRKY81	wild tomato	related with H_2_O_2_ and SA	positive	Wang et al., 2023b
*Blumeria graminis* f. sp. hordei	HvWRKY2	barley	HvCEBi, related with chitin	negative	Yu et al., 2023
*Blumeria graminis* f. sp. tritici	TaWRKY76-D	wheat	Pm2b	negative	Jin et al., 2022
*Penicillium digitatum*	CsWRKY23	citrus	CsAAE12, CsRbohD, CsSARD1, CsWRKY22 and CsIQM6; related with SA, ROS	positive	Wang et al., 2023c
	CsWRKY25	citrus	RbohB, RbohD, and PR10, related with ROS	positive	Wang et al., 2021a
	CsWRKY65	citrus	CsRbohB, CsRbohD, CsCDPK33, and PR10, related with ROS	positive	Wang et al., 2021b
	CsWRKY70	citrus	SAMT, related with SA	positive	Deng et al., 2020
*Fusarium solani*	MdWRKY75	apple	MdERF114, related with lignin	positive	Liu et al., 2023
	MdWRKY74	apple	MdECHT, related with chitin	positive	Xiang et al., 2022
	PnWRKY9	Panax otoginseng	PnDEFL1, related with JA	positive	Zheng et al., 2022
	ScWRKY3	sugarcane	related with ABA, JA and SA	negative	Wang et al., 2018
*Fusarium oxysporum*	Group IIc WRKYs	cotton	GhMKK2, related with flavonoid	positive	Wang et al., 2022b
	PnWRKY22	Panax notoginseng	related with SA	positive	Ning et al., 2021
	LrWRKY2	Lilium regale	LrCHI2, related with Chitinase	positive	Li et al., 2021
	LrWRKY3	Lilium regale	LrDef1, related with SA and JA	positive	Wang et al., 2022c
*Alternaria alternate*	PsnWRKY70	populus	directly interacts with homologous genes and LRR domain proteins	positive	Wang et al., 2022a
	MdWRKY120	apple		negative	Liu et al., 2022
	NaWRKY3	tobacco	related with JA and ET	positive	Xu et al., 2023
*Botrytis cinerea*	LrWRKY39	Lilium regale	related with SA and JA	positive	Fu et al., 2022
	LrWRKY41a	Lilium regale	related with SA and JA	negative	Fu et al., 2022
	LrWRKY4 LrWRKY12	Lilium regale	related with SA and JA	positive	Cui et al., 2018a
	SlWRKY46	tomato	related with ROS, SA and JA	negative	Shu et al., 2021
	SpWRKY6	tomato	related with ABA, SA and JA	negative	Liu et al., 2016
	FvWRKY50	strawberry	related with JA and defense-related genes	positive	Ma et al., 2021
	HbWRKY40	rubber	increased resistance against *B. cinerea* in Arabidopsis	positive	Yang et al., 2020
	PtrWRKY73	populus trichocarpa	related with PAL4 and SA	negative	Duan et al., 2015
*Puccinia horiana* Henn	CmWRKY15-1	chrysanthemums	CmNPR1, related with PR genes and SA	positive	Gao et al., 2022
*Colletotrichum gloeosporioides*	JrWRKY21	walnut	the PR gene JrPTI5L,	positive	Zhou et al., 2022
*Botrytis cinerea* *Verticillium dahliae*	GbWRKY1	cotton	GhJAZ1, related with JA	negative	Li et al., 2014
*Rhizopus stolonifer*	PpWRKY46 PpWRKY53	peaches	related with JA	positive	Ji et al., 2022
	PpWRKY22	peaches	PpHOS1/PpTGA1, related with SA	positive	Li et al., 2022a
	PpWRKY45	peaches	related with JA and phenylpropanoid	positive	Ji et al., 2021a
	PpWRKY70	peaches	related with JA and phenylpropanoid		Ji et al., 2021b
*Zymoseptoria tritici*	TaWRKY10	wheat	related with JA	negative	Campanaro et al., 2021
*Botryosphaeria dothidea*	MdWRKY15	apple	MdICS1, related with SA	positive	Zhao et al., 2020
*Verticillium dahlia*	GhWRKY41	cotton	GhWRKY41, related with phenylpropanoid	positive	Xiao et al., 2023
	GhWRKY70	cotton	related with ET, JA and SA	negative	Xiong et al., 2019
	GhWRKY1-like	cotton	interacts with *GhPAL6* and *GhCOMT1*	positive	Hu et al., 2021
*Colletotrichum musae*	MaWRKY1/ MaWRKY2	banana	MaNAC5, related with JA and SA	positive	Shan et al., 2016
*Sclerotinia sclerotiorum*	BnWRKY33	oilseed rape	related with JA and SA	positive	Liu et al., 2018a
	BnWRKY15	oilseed rape	interacted with BnWRKY33	negative	Liu et al., 2018a
*Puccinia striiformis*	TaWRKY49	wheat	related with SA, JA, ET and ROS	negative	Wang et al., 2017b
	TaWRKY62	wheat	related with SA, JA, ET and ROS	positive	Wang et al., 2017b
	TaWRKY19	wheat	interacted with TaNOX10	negative	Wang et al., 2022e
*Puccinia triticina*, *Fusarium pseudograminearum*, *Rhizoctonia cerealis*, *Puccinia striiformis* f. sp. Tritici, *Blumeria graminis* f. sp. Tritici	HvWRKY6	barly	SA signaling activated, but JA and ABA repressed	positive	Li et al., 2020; 2022b
*Puccinia striiformis* f. sp.tritici, *Blumeria graminis* f. sp. tritici	HvWRKY70	barly		positive	Li et al., 2020
*Magnaporthe oryzae*	OsWRKY42	rice	related with JA	negative	Cheng et al., 2015
	OsWRKY89	rice	wax content related	positive	Wang et al., 2007
	OsWRKY53	rice	OsMPK3/OsMPK6-mediated phosphorylation	positive	Chujo et al., 2014
	OsWRKY30	rice	LOX, AOS2, PR3 and PR10, related with JA	positive	Peng et al., 2012
	OsWRKY31	Rice	related with JA and SA	negative	Wang et al., 2023d

## Role of WRKY TFs in plant resistance to bacterial pathogens

### Pseudomonas syringae

*Pseudomonas syringae* is a common gram-negative plant bacteria widely present in nature. Diseases caused by *P. syringae* rank the first tier among the plant bacterial diseases and cause huge economic losses to global agricultural production (Lai et al., 2008; Hu et al., 2012; Arrano-Salinas et al., 2018). Many WRKY TFs regulating *P. syringae* resistance have been isolated from Arabidopsis, such as AtWRKY11, AtWRKY17, AtWRKY18, AtWRKY40, AtWRKY60, AtWRKY25, AtWRKY3, AtWRKY4, AtWRKY38, AtWRKY62, AtWRKY46, AtWRKY70, AtWRKY53, and AtWRKY7/11/17 etc (Journot-Catalino et al., 2006; Xu et al., 2006; Zheng et al., 2007; Lai et al., 2008; Kim et al., 2008; Hu et al., 2012; Arrano-Salinas et al., 2018). The functions of these transcription factors have been discussed in previously published articles (Eulgem and Somssich, 2007; Pandey and Somssich, 2009; Amorim et al., 2016; Finatto et al., 2018). Recent studies found that AtWRKY1 negatively regulates Arabidopsis response to *P. syringae* pv. tomato DC3000 (Pto DC3000) by activating the SA signaling pathway and inhibiting the expression of *PR1*, *PR2*, and *PR5* genes (Fang et al., 2021).

In *Ocimum sanctum*, the expression of OscWRKY1 gene was induced by JA, SA, and wound, and OscWRKY1 interacts with the W-box *cis*-element in the promoters of *PAL* and *C4H* to positively regulate phenylpropanoid metabolism, which leads to a change in the content of rosmarinic acid and an ultimate increase in resistance against Pto DC3000 (Joshi et al., 2022). Accordingly, mulberry MiWRKY53 regulates the resistance to Pto DC3000 through activating SA-mediated responses (Negi and Khurana, 2021). On the other hand, AaWRKY17 of *Artemisia annua* directly binds to the W-box motifs in the promoter of artemisinin biosynthetic gene *ADS* (amorpha-4,11-diene synthase) and promotes its expression to increases the artemisinin content, which enhances the resistance to Pto DC3000 (Chen et al., 2021a). Tomato SlWRKY8 increases the expression of pathogen-related genes *SlPR1a1* and *SlPR7* to improve the resistance to Pto DC3000 (Gao et al., 2020). Grape VqWRKY52, known as a positive regulator of powdery mildew resistance, enhances the resistance to Pto DC3000 but increases the susceptibility to *Botrytis cinerea* (Wang et al., 2017a). Populus PtrWRKY73 that negatively regulate the resistance to *Botrytis cinerea* could increase populus resistance to Pto DC3000 (Duan et al., 2015). These phenomena indicate that WRKY TFs regulate a broad-spectrum pathogen resistance in plants.

### Ralstonia solanacearum

**Figure 2:**
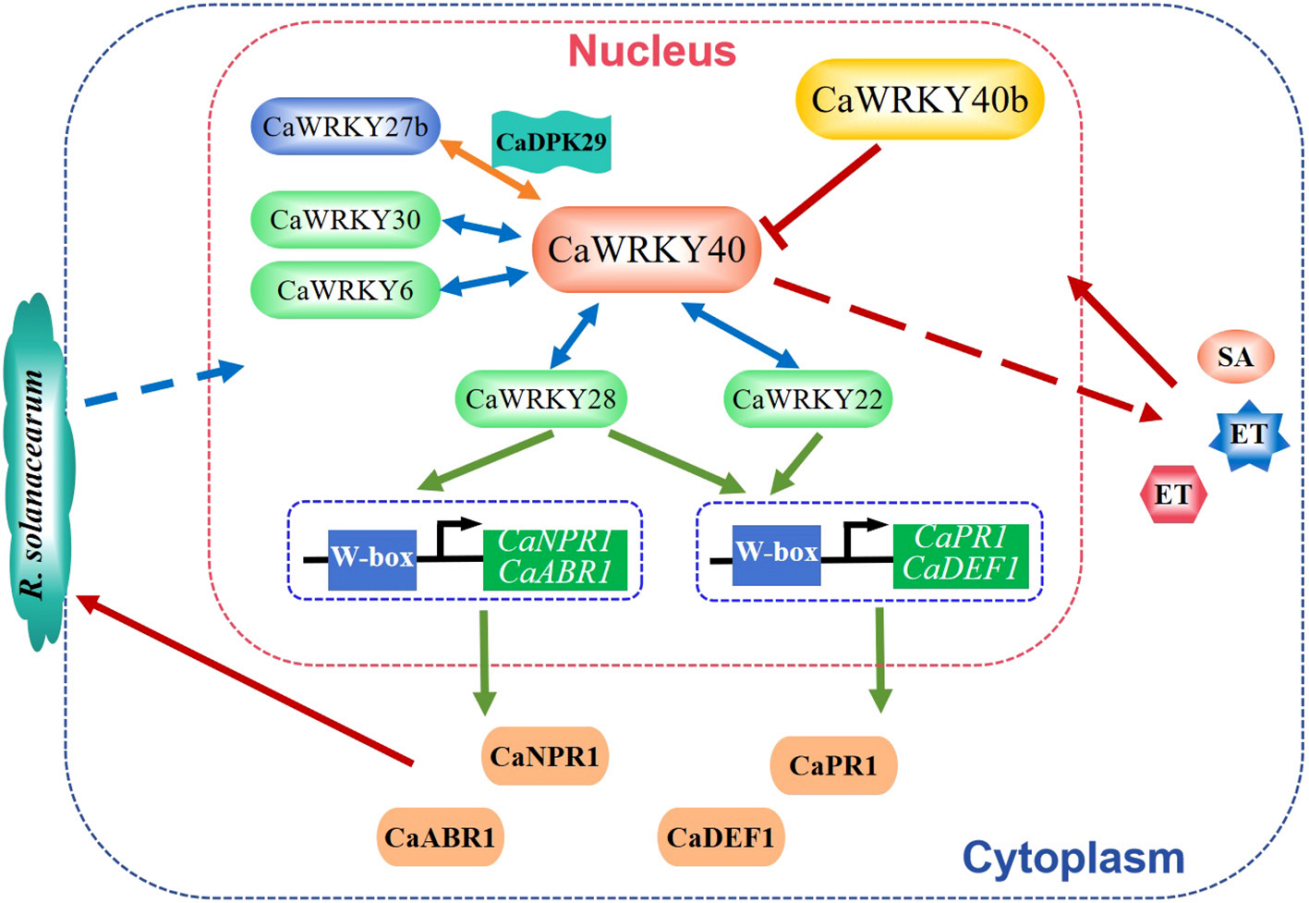
WRKY factors regulate pepper resistance against *Ralstonia solanacearum.* CaWRKY40 could interact with CaWRKY22, CaWRKY28, CaWRKY27b, CaWRKY30, and CaWRKY6 and affect the SA, JA and ET signaling to positively regulate pepper resistance to *R. solanacearum*. Yet, the function of CaWRKY40 is suppressed by CaWRKY40b (Dang et al., 2013; Khan et al., 2018; Yang et al., 2021a, 2021; Hussain et al., 2022).

Bacterial wilt is a devastating soil-borne plant disease caused by *Ralstonia solanacearum*. So far, eight WRKY TFs related to bacterial wilt resistance have been isolated from chili peppers, including CaWRKY40, CaWRKY28, CaWRKY27b, CaWRKY30, CaWRKY6, CaWRKY27, CaWRKY22, and CaWRKY40b. Among them, CaWRKY40 is the first identified WRKY TF. Overexpression of CaWRKY40 enhanced the resistance to *R.solanacearum* in tobacco, while silencing of CaWRKY40 enhanced the susceptibility to *R. solanacearum* in pepper (Dang et al., 2013). CaWRKY40 is regulated by SA, JA and ET signaling and coordinates the responses mediated by these phytohormones to *R. solanacearum* (Dang et al., 2013). The other seven WRKY TFs, except CaWRKY40b, positively regulate pepper resistance to *R. solanacearum*. CaWRKY28 functions not by direct modulating the W-box containing immunity-related genes but by promoting the binding of CaWRKY40 to the immunity-related target genes, including *CaPR1*, *CaNPR1*, *CaDEF1*, and *CaABR1*, to activate their expression (Yang et al., 2021a). Similarly, CaWRKY27b is phosphorylated by CaCDPK29 and acts as a transcriptional activator of CaWRKY40 to initiate the response to *R. solanacearum* (Yang et al., 2021b). CaWRKY30 and CaWRKY6 also function in the *R. solanacearum* resistance by targeting CaWRKY40 (Cai et al., 2015; Hussain et al., 2022). Meanwhile, CaWRKY22 is regulated by SA, JA, and ET and directly binds to the promoters of *CaPR1*, *CaDEF1* (a defense-related gene), and *CaWRKY40* to controls host pathogen resistance (Hussain et al., 2018). Consistently, CaWRKY27 positively regulates tobacco resistance to *R. solanacearum* and its function is associated with the SA, JA, and ET mediated signaling (Dang et al., 2014). On the contrary, CaWRKY40b is a homolog of AtWRKY40 and negatively regulates pepper immunity against *R. solanacearum* (**Figure 2**) via the transcriptional modulation of a subset of immunity-associated genes as well as *CaWRKY40* (Khan et al., 2018).

In cotton, GhWRKY44 plays a positive role in regulating resistance to *R. solanacearum* infection. Overexpression of *GhWRKY44* in cotton induced the expression of several defense-related genes, including *PR-1*, *PR-2*, *PR-5*, and *NPR1* for SA signaling and *PR-4* for JA signaling, to increase *R. solanacearum* resistance (Li et al., 2015c). ScWRKY3, a negative regulator of *F. solani* infection, also plays a negative regulation during the challenges by bacterial pathogen *R. solanacearum* (Wang et al., 2018). In 2022, 174 WRKY genes were identified from the genome of cultivated peanuts. Among the peanut WRKY TFs, 16 members were found to be upregulated by *R. solanacearum* infection, indicating an importance of these transcription factors in regulating peanut resistance (Yan et al., 2022b).

### *Xanthomonas oryzae* pv. *oryzae*

**Figure 3:**
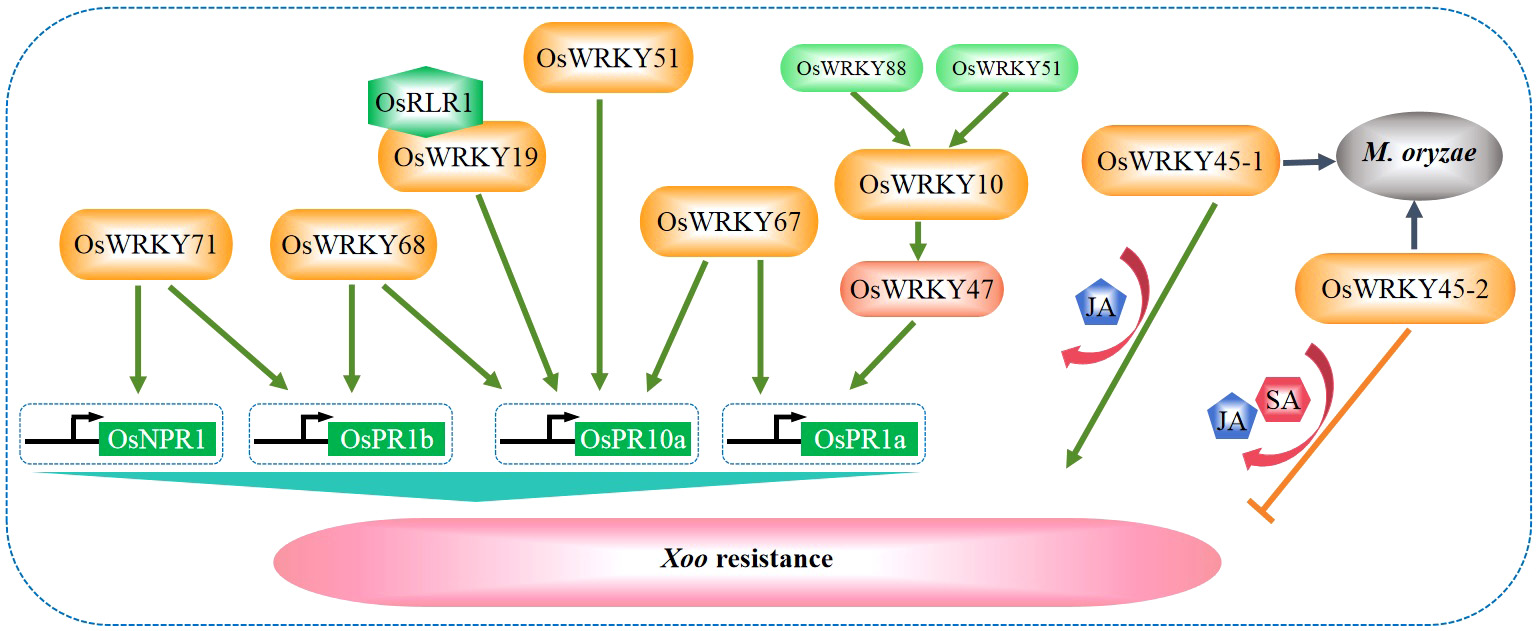
WRKY factors regulate rice resistance against *Xanthomonas oryzae* pv*. Oryzae* (*Xoo*). Rice WRKY TFs regulate the resistance to *Xoo* by directly modulating the expression of defense-related genes, mediating the phytohormone signaling of JA and SA, or interacting with other regulators including OsRLR1 (Tao et al., 2009; Yang et al., 2016; Liu et al., 2018b; Choi et al., 2020; Du et al., 2021).

Bacterial blight caused by *Xanthomonas oryzae* pv*. oryzae* (*Xoo*) is a major disease resulting in significant losses to rice production. Currently, multiple WRKY TFs related to bacterial blight have been isolated from rice, including OsWRKY10, OsWRKY11, OsWRKY13, OsWRKY19, OsWRKY45-1, OsWRKY45-2, OsWRKY47, OsWRKY51, OsWRKY53, OsWRKY62, OsWRKY67, OsWRKY68, OsWRKY71, and OsWRKY88. Two transcriptional regulatory cascades have been established in OsWRKY10-mediated blight disease resistance. In the first transcriptional regulatory cascade, OsWRKY47 acts downstream of OsWRKY10, while OsWRKY51 acts upstream of OsWRKY10 (Choi et al., 2020). In the second transcriptional regulatory cascade, OsWRKY47 acts downstream of OsWRKY10, and OsWRKY88 acts upstream of OsWRKY10. Specifically, OsWRKY10 binds to the promoter of *OsPR1a* to activate its expression and positively regulates the resistance to *X. oryzae* (Choi et al., 2020) as illustrated in **Figure 3**.

During the interaction between rice and *Xoo*, OsWRKY68 is induced and binds to the W-boxes in the promoters of *OsPR1b* and *OsPR10a* to activate their expression (Yang et al., 2016). In contrast, OsWRKY71 functions as a transcriptional regulator upstream of OsNPR1 and OsPR1b and positively regulates the defense response against *Xoo* (Liu et al., 2007; **Figure 3**). OsWRKY51 also serves as a positive regulator to directly bind to the promoter of *OsPR10a* (Hwang et al., 2016). Recently, it was evidenced that the OsRLR1 (RPM1-like resistance gene 1)-OsWRKY19-OsPR10 complex plays a positive role in regulating *Xoo* resistance (Du et al., 2021). In addition, OsWRKY67 positively regulates rice responses to leaf blast, panicle blast, and bacterial blight. It activates the expression of *OsPR1a* and *OsPR10* by directly binding to their promoters and induces the expression of various other defense-related genes (**Figure 3**), including those involved in the SA-dependent pathway (Liu et al., 2018b).

Two allelic genes *OsWRKY45-1* and *OsWRKY45-2*, whose encoding proteins differ for 10 amino acids, play opposite roles in regulating rice resistance against *Xoo*. OsWRKY45-1 negatively regulates rice resistance to *Xoo*, while OsWRKY45-2 positively regulates the resistance (Tao et al., 2009). The OsWRKY45-1-mediated *Xoo* resistance is accompanied by increased accumulation of SA and JA and enhanced expression of a subset of defense-responsive genes. On the other hand, the OsWRKY45-2-mediated regulation of *Xoo* resistance is accompanied by increased accumulation of JA (but not SA) and enhanced expression of another subset of defense-responsive genes (Tao et al., 2009; **Figure 3**). Interestingly, both OsWRKY45-1 and OsWRKY45-2 are positive regulators in rice resistance against *M. grisea*.

Rice OsMYB63 promotes the expression of secondary cell wall-related cellulose synthase genes and the accumulation of cellulose, which results in thickened sclerenchyma cell walls, to provide resistance against pathogen infection (Xie et al., 2021). However, OsWRKY53 acts upstream of *OsMYB63* to suppress its expression thus to negatively regulate the resistance to *Xoo* by weakening the sclerenchyma cell walls of vascular bundle (Xie et al., 2021). OsWRKY11 is also known to initiate defense responses to *Xoo* and it directly binds to the promoter of chitinase 2-encoding defense gene to activate its expression (Lee et al., 2018).

The WRKY TFs identified to regulate plant resistance to bacterial pathogens are listed in **Table 2**.

**Table 2:** The list of WRKY TFs that regulate plant responses to bacterial pathogens.

Pathogens	Names	Species	Target proteins	Functions	References
*P. syringae* pv. tomato DC3000	AtWRKY1	Arabidopsis	SARD1 and CBP60g, related with PR1 and SA	negative	Fang et al., 2021
	OscWRKY1	Ocimum sanctum	PAL and C4H, related with rosmarinic acid and SA	positive	Joshi et al., 2022
	MiWRKY53	mulberry	related with PR1 and SA		Negi and Khurana, 2021
	AaWRKY17	Artemisia annua	ADS, related with artemisinin content	positive	Chen et al., 2021a
	SIWRKY8	tomato	related with SlPR1a1 and SlPR7	positive	Gao et al., 2020
*Ralstonia solanacearum*	CaWRKY40	pepper	related with SA, JA and ET	positive	Dang et al., 2013
	CaWRKY28	pepper	promoting the binding of CaWRKY40 with target genes	positive	Yang et al., 2021a
	CaWRKY27b	pepper	a transcriptional activator of CaWRKY40	positive	Yang et al., 2021b
	CaWRKY30	pepper	CaWRKY40	positive	Hussain et al., 2022
	CaWRKY6	pepper	CaWRKY40	positive	Cai et al., 2015
	CaWRKY22	pepper	CaWRKY40, CaPR1, and CaDEF1	positive	Hussain et al., 2018
	CaWRKY27	pepper	related with SA, JA and ET	positive	Dang et al., 2014
	CaWRKY40b	pepper	targets a number of immunity- associated genes, including CaWRKY40	negative	Khan et al., 2018
	GhWRKY44	cotton	related with JA and SA	positive	Li et al., 2015c
	ScWRKY3	sugarcane	related with ABA, JA and SA	negative	Wang et al., 2018
*Xanthomonas* *oryzae* pv. oryzae	OsWRKY10	rice	OsPR1a, OsWRKY47, OsWRKY88, OsWRKY51	positive	Choi et al., 2020
	OsWRKY51	rice	related with OsPR10a	positive	Hwang et al., 2016
	OsWRKY68	rice	related with PR1b and PR10a		Yang et al., 2016
	OsWRKY71	rice	OsNPR1 and OsPR1b	positive	Liu et al., 2007
	OsWRKY19	rice	related with OsRLR1 and OsPR10	positive	Du et al., 2021
	OsWRKY67	rice	related with PR1a, PR10 and SA	positive	Liu et al., 2018b
	OsWRKY45-1	rice	related with SA and JA	negative	Tao et al., 2009
	OsWRKY45-2	rice	related with JA	positive	Tao et al., 2009
	OsWRKY53	rice	related with OsMYB63 and sclerenchyma	negative	Xie et al., 2021
	OsWRKY11	rice	related with CHITINASE2	positive	Lee et al., 2018

## Role of WRKY TFs in plant resistance to oomycete pathogens

*Pseudoperonospora cubensis*, *Hyaloperonospora parasitica* and *Phytophthora* spp. are the major oomycete pathogens affecting plants. Various WRKY TFs have been identified to regulate plant responses to these oomycetes. In cucumber, CsWRKY50 positively regulates the resistance to *H. parasitica* via multiple signaling pathways to activate the expression of SA- and JA-responsive genes as well as the SA biosynthetic genes (Luan et al., 2019). In Arabidopsis, AtWRKY33 negatively regulates the resistance to *H. parasitica* and its gene expression is modulated by PAD4, a key component upstream of the SA signaling (Lippok et al., 2007 *)*. In addition, AtWRKY70 performs a positive role in the RPP4 (recognition of *H. parasitica*)-mediated resistance and basal defense against *H. parasitica* under the regulation by PAD4 and SA signaling (Knoth et al., 2007). In *Solanum pimpinellifolium*, SpWRKY1 and SpWRKY3 positively regulate the response to *P. infestans*. SpWRKY1 enhances the expression of ROS scavenging-related genes, SA/JA-responsive genes, and SA/JA biosynthetic genes (Li et al., 2015a; Cui et al., 2019a), while SpWRKY3 induces the expression of *PR* genes and reduces ROS accumulation to protect cell membrane from injury and to enhance the resistance (Cui et al., 2018b). SpWRKY1 also positively regulates the resistance to *P. nicotianae* via the SA and JA signaling (Li et al., 2015b). In soybean, GmWRKY40 functions as a positive regulator of plant response to *P. sojae* by modulating the JA signaling and hydrogen peroxide accumulation (Cui et al., 2019b)

The WRKY TFs identified to regulate plant resistance to oomycete pathogens are listed in **Table 3**.

**Table 3:** List of WRKY TFs that regulate plant responses to oomycete pathogens.

Pathogens	Names	Species	Targets	Functions	References
*Pseudoperonospora cubensis*	CsWRKY50	cucumber	related with SA, JA	positive	Luan et al., 2019
*Hyaloperonospora* *parasitica*	AtWRKY33	Arabidopsis	Related with PAD4, but independent of SA	negative	Lippok et al., 2007
	AtWRKY70	Arabidopsis	related with PAD4, SA	positive	Knoth et al., 2007
*Phytophthora infestans*	SpWRKY1	currant tomato	related with ROS, JA and SA	positive	Li et al., 2015a
	SpWRKY3	currant tomato	related with PR gene and ROS	positive	Cui et al., 2018b
*Phytophthora nicotianae*	SpWRKY1	currant tomato	related with JA and SA	positive	Li et al., 2015b
*Phytophthora sojae*	GmWRKY40	soybean	related with JA and ROS	positive	Cui et al., 2019b

## Role of WRKY TFs in plant resistance to viruses and other pathogens

Studies have demonstrated the role of WRKY TFs in regulating plant resistance to viruses. CaWRKY-a of *Capsicum annuum*, NbWRKY1 of tobacco, and group III WRKY TFs of tomato regulate the resistance against tobacco mosaic virus, mulberry mosaic dwarf virus, and tomato yellow leaf curly virus (TYLCV), respectively (Park et al., 2006; Sun et al., 2022b; Huang et al., 2016), however, the detailed mechanisms underlying the function of these factors remain unclear.

Soybean is one of the plant species with the largest WRKY gene family, and at least three WRKY factors, including GmWRKY136, 53, and 86, have been identified to positively regulate the resistance to soybean cyst nematode (SCN; *Heterodera glycines*), which is the most devastating pathogen to soybean (Yang et al., 2017). Accordingly, wheat TaWRKY74 positively regulates the resistance to ‘*Candidatus* Phytoplasma tritici’ (‘Ca. P. tritici’), but it is destabilized by the pathogen effector SWP12, which weakens plant resistance and promotes virus colonization (Bai et al., 2022). Tomato SlWRKY45 could interact with most JA-ZIM domain family JAZ proteins, key repressors of the JA signaling, to attenuate JA biosynthesis and to repress the defense against root-knot nematodes (Huang et al., 2022).

## Mechanisms underlying the regulation of plant immunity by WRKY factors

Numerous WRKY TFs have been identified to regulate plant resistance against pathogens. These WRKY factors can regulate plant pathogen resistance via multiple pathways, mainly involving the action of phytohormones, MAPK (mitogen-activated protein kinases) signaling cascades, and other signaling pathways.

**Figure 4:**
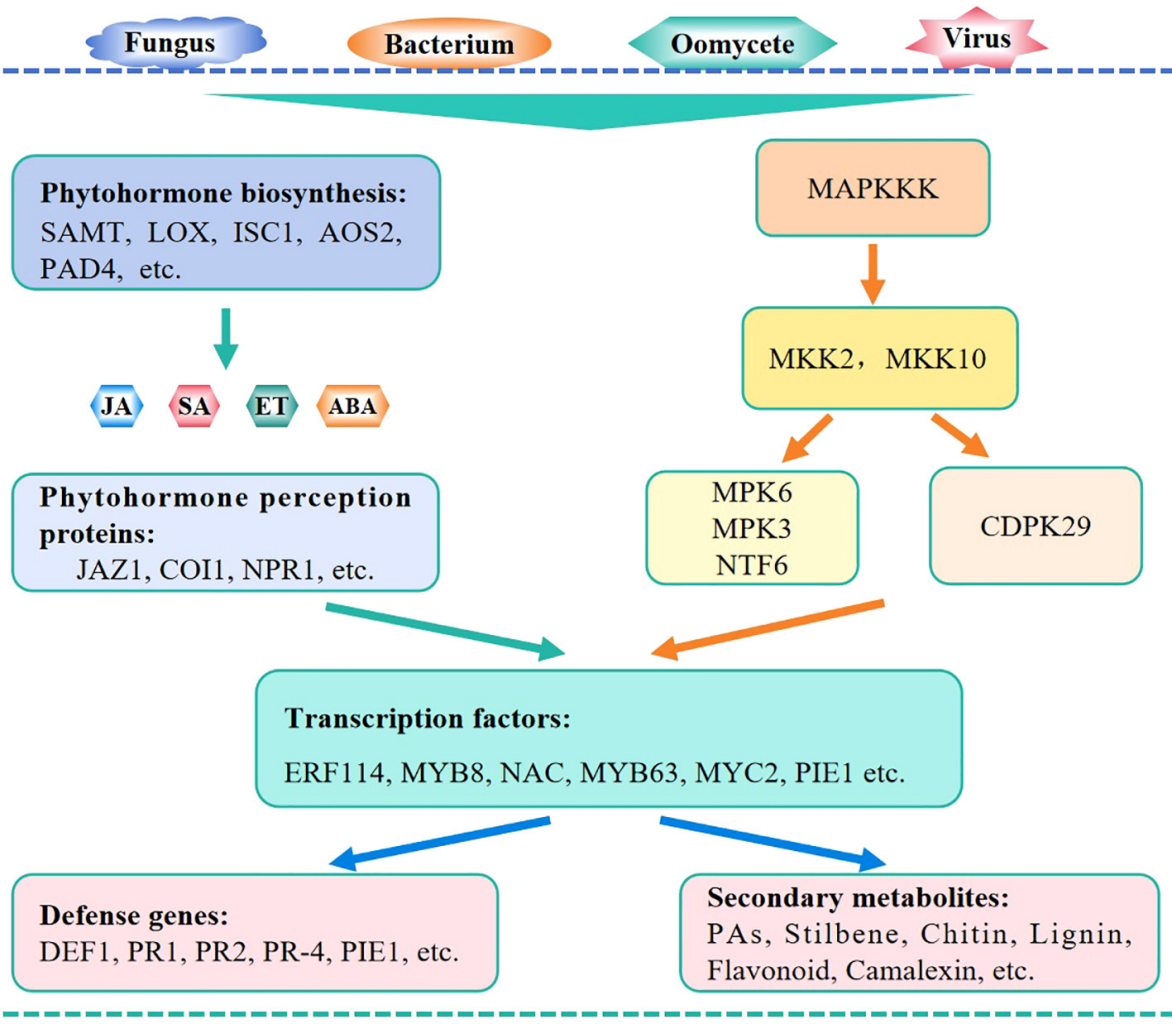
Mechanism module of WRKY TFs in regulating plant immunity against pathogens. WRKY TFs regulate plant pathogen resistance mainly via the action of phytohormone signaling, MAPK cascades, and other signaling pathways. During these processes, WRKY TFs may modulate the expression of phytohormone biosynthetic genes, affect the components in phytohormone signaling and MAPK cascades, or interact with other regulators including WRKY TFs.

In plants, phytohormones such as SA, JA, ABA, and ET are essential regulators of immunity. The WRKY factors specifically regulate the biosynthesis and signaling pathways of these phytohormones to modulate the expression of downstream disease resistance-related genes (**Figure 4**). For example, CsWRKY70 of citrus and MdWRKY15 of apple activate the expression of SAMT and MdICS1- encoding genes, respectively, to regulate plant pathogen resistance (Deng et al., 2020; Zhao et al., 2020). Additionally, OsWRKY30, OsWRKY42, PpWRKY45, PpWRKY70, and LrWRKY3 are involved in the biosynthesis of SA and JA, which in turn changes the plant resistance levels.

NPR1 is a master regulator of the SA signaling pathway, while COI1 and JAZ are pivotal components of the JA signaling pathway. All these components are targets of WRKY TFs. Specifically, CmWRKY15-1, GhWRKY44, and OsWRKY71 function as transcriptional regulators upstream of NPR1 to regulate the plant defense response against pathogens. Moreover, TaWRKY10 and GbWRKY1 regulate the expression of genes encoding the JA-signaling components TaCOI1 and GhJAZ1, respectively, to regulate plant pathogen resistance. Among the WRKY TFs discussed in this article, almost 99% of them are associated with the JA and SA signaling, which is accompanied with changes in ROS accumulation and *PR* gene expression. In addition, other phytohormones, such as ET and ABA, are involved in the WRKY TFs-assisted immune network in regulating plant resistance against pathogens (Dang et al., 2014; Hussain et al., 2018; Wang et al., 2018).

Furthermore, WRKY TFs participate in the MAPK signaling pathways. MAPK signaling cascades link upstream receptors to downstream transcription factors via multiple phosphorylation events to function in the defensive responses in plants (Jagodzik et al., 2018). Group IIc WRKY TFs in cotton and PsnWRKY70 in *Populus nigra* both activate the MAP kinase cascade genes to enhance host resistance against pathogens. In tomato infected by *F. oxysporum*, the genes encoding MAPK and WRKY regulators are majorly enriched in the metabolic pathways related to disease resistance (Sun et al., 2022a). OsWRKY53 is activated by the OsMPK3/OsMPK6-mediated phosphorylation in the regulation of blast resistance (Chujo et al., 2014). OsWRKY31 is a key component in the MAPK signaling pathway involved in rice resistance to *M. oryzae* and is modulated by phosphorylation and ubiquitination via RING-finger E3 ubiquitin ligases (Wang et al., 2023d). CaWRKY27b is phosphorylated by CaCDPK29 to act as a transcriptional activator of CaWRKY40 and to initiate the response to *R. solanacearum* (Yang et al., 2021b).

Additionally, WRKY TFs may interact with other transcription factors to regulate plant pathogen resistance. The transcription factor complexes, such as MdWRKY75-MdERF114-MdMYB8-MdPRX63, MaNAC5-MaWRKY1-MaWRKY2, BnWRKY15-BnWRKY33, and CaWRKY40-CaWRKYs play important roles in regulating host plant resistance.

## Conclusions and future perspectives

Plants employ multiple approaches to defend pathogen attack during their development processes, among which the WRKY TFs play crucial roles. Since the discovery of the first WRKY TF, lots of studies on the identification and function dissection of WRKY TFs have been reported (**Supplementary Table S1**). Recent studies showed that WRKY TFs coordinate a sophisticated network composed of phytohormone signaling, MAPK pathway, protein interaction, regulation of functional genes, and other immune responses in reprogramming plant responses against pathogens. WRKY TFs may exhibit either positive or negative role in regulating plant resistance to pathogens. Even though the majority of the identified WRKY TFs display positive functions, some of them show negative roles during plant responding to pathogen attack. For instance, 63 of the WRKY TFs reviewed in this article are positive regulators, whereas 21 of them are negative regulators. Furthermore, some WRKY TFs could mediate plant immunity to a broad spectrum of pathogens, such as HvWRKY6 regulating barley resistance to Pst, Bgt, Pt, Fpg, and Rc (Li et al., 2020; 2022b). Therefore, the function of WRKY TFs in regulating plant pathogen resistance is diverse and complex.

Plant genomes contain massive WRKY TFs to be investigated. The developing high-throughput transcriptomics, proteomics, metabolomics, gene editing and biosynthetic technologies will provide more efficient approaches to the identification and functional analysis of WRKY TFs. The function of WRKY TFs should be thoroughly revealed by future in-depth studies, and the mechanism underlying their regulation of pathogen resistance will be comprehensively understood. Thus, future studies may provide good choices for WRKY TFs of negative roles in regulating plant pathogen resistance to be targeted for gene editing or RNAi and those of positive roles in this process to be targeted for overexpression to improve plant disease resistance.
