# Supplementary material for: Recent advances in functional assays of WRKY transcription factors in plant immunity against pathogens
Source: Front Plant Sci. 2025 Jan 23;15:1517595. doi: 10.3389/fpls.2024.1517595 (PMC11798884; doi:10.3389/fpls.2024.1517595)
Supplement: Supplementary file 1 [file Table1.docx]

**Supplementary Table**

**Table S1 The WRKY numbers in different species**

|  | **Species** | **Members of the**  **WRKY gene family** | | **References** |
| --- | --- | --- | --- | --- |
| Non-plants | *Giardia lamblia* | | 1 | ULker and Somssich., 2004 |
|  | *Dictyostelium discoideum* | 1 | | Zhang and Wang., 2005 |
|  | *Dictyostelium purpureum* | 9 | | Mohanta et al., 2016 |
| Algae | *Coccomyxa subellipsoidea* | 1 | | Mohanta et al., 2016 |
|  | *Klebsormidium flaccidum* | 2 | | Hori et al., 2014 |
|  | *Micromonas pusilla* | 2 | | Mohanta et al., 2016 |
|  | *Ostreococcus lucimarinus* | 2 | | Mohanta et al., 2016 |
|  | *Ostreococcus tauri* | 3 | | Rinerson et al., 2015 |
|  | *Physcomitrella patens* | 41 | | Rensing, et al., 2008 |
|  | *Volvox carteri* | 2 | | Mohanta et al., 2016 |
| Flowering plants | *Akebia trifoliate* | 63 | | Zhu et al., 2022 |
|  | *Amborella moellendorffii* | 19 | | Li et al., 2016 |
|  | *Aquilegia coerulea* | 33 | | Mohanta et al., 2016 |
|  | *Arabidopsis lyrata* | 79 | | Mohanta et al., 2016 |
|  | *Arabidopsis thaliana* | 74 | | Mohanta et al., 2016 |
|  | *Musa nana Lour* | 287 | | Kaliyappan et al., 2016 |
|  | *Ananas comosus* L | 54 | | Xie et al., 2018 |
|  | *Arachis hypogaea* L | 174 | | Yan et al., 2022b |
|  | *Brachypodium distachyon* | 81 | | Mohanta et al., 2016 |
|  | *Brassica rapa* | 145 | | Mohanta et al., 2016 |
|  | *Brassica napus* | 153 | | He et al., 2016 |
|  | *Capsella rubella* | 72 | | Mohanta et al., 2016 |
|  | *Carica papaya* | 50 | | Mohanta et al., 2016 |
|  | *Caragana intermedia* L | 97 | | Wan et al., 2018 |
|  | *Chinese cabbage* | 148 | | Yan et al., 2022a |
|  | *Citrus clementina* | 52 | | Mohanta et al., 2016 |
|  | *Citrus sinensis* | 92 | | Li et al., 2016 |
|  | *Cucumis sativus* | 62 | | Mohanta et al., 2016 |
|  | *Cucumis melo* L | 57 | | Chen et al., 2021b |
|  | *Daucus carota* | 95 | | Li et al., 2016 |
|  | *Eucalyptus grandis* | 78 | | Mohanta et al., 2016 |
|  | *Fragaria vesca* | 56 | | Mohanta et al., 2016 |
|  | *Glycine max L. Merr* | 174 | | Yang et al., 2017 |
|  | *Gossypium raimondii* | 120 | | Mohanta et al., 2016 |
|  | *Hevea brasiliensis* | 111 | | Yang et al., 2020 |
|  | *Hordeum vulgare* | 45 | | Mangelsen et al., 2008 |
|  | *Jatropha curcas* | 58 | | Finatto et al., 2018 |
|  | *Juglans regia* L | 103 | | Hao et al., 2021 |
|  | *Linum usitatissimum* | 105 | | Mohanta et al., 2016 |
|  | *Lilium regale Wilson* | 25 | | Fu et al., 2022 |
|  | *Malus domestica* | 139 | | Li et al., 2016 |
|  | *Manihot esculenta* | 117 | | Finatto et al., 2018 |
|  | *Medicago truncatula* | 76 | | Mohanta et al., 2016 |
|  | *Mimulus guttatus* | 65 | | Mohanta et al., 2016 |
|  | *Morus notabilis* | 54 | | Finatto et al., 2018 |
|  | *Nelumbo nucifera* | 62 | | Li et al., 2016 |
|  | *Nicotiana tabacum*L*.* | 93 | | Rushton et al., 2008 |
|  | *Oryza sativa indica* | 116 | | Finatto et al., 2018 |
|  | *Oryza  japonica* | 137 | | Finatto et al., 2018 |
|  | *Oryza nivara* | 97 | | Xu et al., 2016 |
|  | *Panicum hallii* | 97 | | Mohanta et al., 2016 |
|  | *Panicum virgatum* | 168 | | Mohanta et al., 2016 |
|  | *Panax notoginseng (Burk) F.H.Chen* | 30 | | Zheng et al., 2022 |
|  | *Phaseolus vulgaris* | 88 | | Mohanta et al., 2016 |
|  | *Phoenix dactylifera* | 78 | | Li et al., 2016 |
|  | *Physcomitrella patens* | 41 | | Li et al., 2016 |
|  | *Picea abies Gymnosperm* | 62 | | Mohanta et al., 2016 |
|  | *Picea abies* | 72 | | Li et al., 2016 |
|  | *Pinus monticola* | 84 | | Liu and Ekramoddoullah, 2009 |
|  | *Populus trichocarpa* | 102 | | Mohanta et al., 2016 |
|  | *Phaseolus vulgaris* L | 88 | | Wu et al., 2017 |
|  | *Prunus persica* | 60 | | Mohanta et al., 2016 |
|  | *Ricinus communis* | 58 | | Li et al., 2016 |
|  | *Rusclementina* | 81 | | Finatto et al., 2018 |
|  | *Selaginella moellendorffii* | 19 | | Mohanta et al., 2016 |
|  | *Setaria italica* | 106 | | Mohanta et al., 2016 |
|  | *solanum lycopersicum* | 83 | | Karkute et al., 2018 |
|  | *Solanum tuberosum* | 82 | | Liu et al., 2017 |
|  | *Sorghum bicolor* | 90 | | Mohanta et al., 2016 |
|  | *Thellungiella halophila* | 66 | | Mohanta et al., 2016 |
|  | *Theobroma cacao* | 59 | | Mohanta et al., 2016 |
|  | *Vitis vinifera* | 98 | | Finatto et al., 2018 |
|  | *Zea mays* | 180 | | Finatto et al., 2018 |
